# Preprocessing and Capability–Risk Coupling in an Event-Synchronized Dual-Frontend Speech Sensor System

**DOI:** 10.3390/s26185794

**Published:** 2026-09-12

**Authors:** Hansheng Chen, Zhanyu Zhu

**Affiliations:** School of Electronic and Information Engineering, Soochow University, Suzhou 215006, China; 2428403077@stu.suda.edu.cn

**Keywords:** intelligent acoustic sensor, sensor interface, event-synchronized sensing, signal preprocessing, embedded speech recognition, speech-to-action system, capability–risk coupling, runtime assurance

## Abstract

An intelligent speech sensor is often evaluated at recognition output, although acquisition and preprocessing also determine what reaches control. We examine this propagation in an event-synchronized dual-frontend system. A conditional cascade separates artifact availability, evidence formation, semantic conversion, and executor persistence. It decomposes how scheduled intent survives to the action boundary from raw audio through controlled motion. Paired recordings compare an INMP441–ESP32 interface with a USB interface. In an exploratory study, five speakers each delivered 30 Chinese utterances in three acoustic conditions, giving 450 scheduled events, 1800 ASR paths, and 3600 decision paths. Twelve missing artifacts remained in the intention-to-test denominators. Under the frozen peak normalization rule, isolated full-scale INMP samples limited window gain, and normalized USB–INMP speech-band differences reached +20.96 to +29.52 dB. Mean cloud text availability was 48.0 percentage points higher on USB. Exact valid execution rose by 41.7 points for DeepSeek and by 26.7 points for MiMo. Unauthorized non-stop motion on reject events rose by 19.6 points for DeepSeek and by 13.0 points for MiMo. Capability and risk increased together in all ten cloud speaker–consumer contrasts. Among 564 dangerous reject paths, 340 contained no literal motion token. In the worst case per speaker, hold-last replay produced 72.2–100% dangerous reject states. This is an exploratory sensor system case study of the tested frontend and preprocessing configuration, not a capsule ranking. Preprocessing changed useful evidence reach and action opportunity, so coverage, authorization, and acknowledged executor state need joint evaluation. The empirical results are specific to the tested pipeline and the five observed speakers.

## 1. Introduction

An intelligent acoustic sensor does more than convert sound into a waveform. In embedded speech control, acquisition, transport, preprocessing, recognition, language interpretation, and execution form one sensing system. Recent studies cover this chain through speech interfaces, edge assistants, acoustic nodes, and robot platforms [1,2,3,4,5,6,7]. A change near the microphone can therefore travel far enough to alter the state a controller reaches.

Many acoustic interface evaluations stop at recognition accuracy, latency, keyword detection, or task completion. Embedded keyword spotting and gain control studies show why these measures matter [8,9], but not why individual events fail or whether a null output left an actuator stopped. The two recordings of a pair can diverge at capture, transport, normalization, automatic speech recognition (ASR), semantic authorization, or execution. If every outcome is recorded as “no new command,” lost sensor evidence can look like successful rejection.

This distinction matters when a frontend or its preprocessing is improved. More evidence can increase exact execution, but it also gives rejected utterances another chance to reach the action boundary. A weaker path may appear safer because evidence disappeared earlier. We call the joint increase in useful capability and unauthorized action capability–risk coupling. It belongs to the tested configuration, not to either microphone by itself.

Noise-robust ASR, confidence estimation, weakly supervised recognition, and non-autoregressive decoding address acoustic mismatch and transcription uncertainty [10,11,12,13]. Downstream methods train against ASR errors or retain richer uncertainty representations [14,15,16], while voice benchmarks show that text-input performance does not determine voice-input performance [17]. Embodied language systems can ground instructions, generate action programs, reason through feedback, or ask for help [18,19,20,21,22]. Selective classification and conformal methods formalize abstention [23,24,25,26,27], and runtime assurance separates capable controllers from verified safety logic [28,29,30,31,32]. These strands are individually mature, but they rarely follow a single scheduled spoken event from event-paired sensing to physical-state semantics.

The measurement problem is therefore full-chain. A sensor interface changes the evidence presented to the recognizer. The recognizer then changes what reaches the semantic consumer, which may turn plausible language into a valid-looking action. Finally, the executor decides whether no new action means stop or persistence. A theory that omits any of these boundaries cannot distinguish robust sensing, safe authorization, and accidental silence.

This study combines an event-synchronized paired 3 s protocol, an operational intention-to-test (ITT) ledger, and a conditional sensor-to-action model. Five speakers each completed the same 30-utterance policy set in three acoustic conditions. INMP441–ESP32 and USB paths captured every scheduled event under a common trigger. Here, synchronization denotes event-level pairing of the same spoken window rather than sample-level clock locking. The paired design makes the spoken event, rather than separately recorded sessions, the comparison anchor.

The work makes three connected contributions. A conditional cascade measures evidence reach, capability, reject danger, and executor persistence without assuming independent failures. Event-paired recordings then compare two complete sensing interfaces under the deployed peak normalization rule. Finally, a typed ledger and executor replay connect sensor availability to control state, where semantic abstention counts as a stop only after state clearing and acknowledgement.

The analysis reports speaker-specific paired results before equal-speaker summaries. Repeated ASR and consumer paths from the same event are retained for pipeline accounting, but they are not treated as independent spoken observations.

## 2. Related Work and Sensor System Research Gap

### 2.1. Acoustic Sensor Interfaces and Evidence Formation

Speech-enabled sensor systems combine transducers, acquisition electronics, preprocessing, and inference. This topic has been treated at several scales. One line develops speech and multimodal interfaces, including non-acoustic sensing [2,5,33]. Another builds edge assistants, embedded keyword spotting, and low-cost acoustic sensor nodes [1,9,34,35]. Robot studies then connect speech sensing to interaction, mobility, or emergency behavior [3,4]. Read together, these papers make one point clear: the microphone path and downstream software form an operational interface, even when they are evaluated in separate modules.

Robust ASR research treats acoustic corruption, channel mismatch, and recognition uncertainty as central technical problems [10,11,12,13]. Training-time error augmentation and word-confusion representations can improve downstream language understanding [14,15,16]. More specifically, Wang et al. modeled gain control inside a Mandarin ASR chain and showed that an unsuitable strategy can degrade word error rate [8]. That result is close to the present concern, although our endpoint extends beyond recognition. Capture and service loss remain outcomes, and preprocessing is kept inside the tested sensing chain because it changes the waveform presented to the recognizer.

The acoustic sensor literature also gives a useful caution about hardware labels. Low-cost USB microphones, MEMS microphones, edge processors, and network transport are usually selected and evaluated as complete sensing nodes [34,35]. This is why the present comparison is phrased as INMP441–ESP32 versus USB acquisition pipelines. It is not presented as a capsule-only experiment.

### 2.2. Language-to-Action Mapping and Selective Execution

Language-conditioned robotics demonstrates that broad linguistic evidence can be converted into affordances, code, plans, or help-seeking behavior [18,19,20,21,22]. Reject-option classification and conformal risk methods provide mathematical foundations for withholding a prediction when confidence or calibrated risk is unacceptable [23,24,25,26,27]. These ideas motivate selective execution, but the gate must be defined at the action boundary. A small valid action grammar constrains syntax; it does not prove that the sensed utterance was an authorized invocation of the action.

Speech-driven robotic systems commonly connect recognition output to motion or task modules [2,3,4]. Recent studies have used speech and LLM planning to improve task interpretation and execution efficiency in modular robots [6] and have evaluated multimodal agentic interfaces in real human–robot assembly tasks [7]. Those systems establish feasibility and task performance; the present work addresses a different endpoint: how a paired sensing difference propagates into authorized and unauthorized actions and persistent executor state. Recognition success, semantic acceptance, command emission, and stopped physical state are therefore kept distinct. A frontend that loses more evidence can reduce both useful motion and unauthorized motion. Reporting only the latter would reward sensing failure.

### 2.3. Runtime Assurance and Conditional Reliability

Runtime assurance architectures separate a capable but unverified controller from supervisory logic and a verified safety controller [29,30]. Industrial robot and emergency-stop standards similarly require explicit safety functions and state transitions [31,32]. Reliability block diagrams often express a series path as a product of component reliabilities [36]. A speech-sensing cascade, however, contains nested transitions. ASR is attempted only after an artifact exists, and a semantic consumer is called only after text exists. Executor behavior also depends on the preceding state. The framework below therefore uses the probability chain rule and stage-conditional factors, rather than an independence approximation.

### 2.4. Research Position

The closest studies establish separate parts of this problem. Gain control work connects signal chain design to ASR error [8]; acoustic node studies emphasize complete interfaces and edge processing [34,35]; speech-driven robots connect recognized language with planning and motion [6,7]. Less visible is the propagation between these layers. The question here is how a paired sensing and preprocessing change alters evidence reach, authorized capability, unauthorized action, and executor state.

The complete acquisition and preprocessing interface is the comparison unit here, so the bare microphone capsule is not assigned an intrinsic property. The framework connects five objects that are often reported separately: scheduled intent, sensor evidence reach, semantic authorization, generated action, and executor state. Each analytical statement is paired with an observation that could disagree with it. This arrangement keeps the mathematics close to the sensor experiment and also separates accounting identities from findings that depend on the observed configuration.

## 3. Sensor-to-Action Measurement Framework

### 3.1. Policy Sets, Sensing Chain, and Endpoints

The frozen product-policy puts the utterances into two sets. Authorized utterances belong to V, while utterances that should be rejected belong to R; therefore, V∩R=∅. The executable actions are A={forward,backward,left,right,stop}. When u∈V, the map π(u) specifies the required action sequence; when u∈R, any motion other than stop is unauthorized. The expected answer is fixed before ASR starts, so a fluent but incorrect transcript cannot change the intended command after the fact.

**Figure 1:**
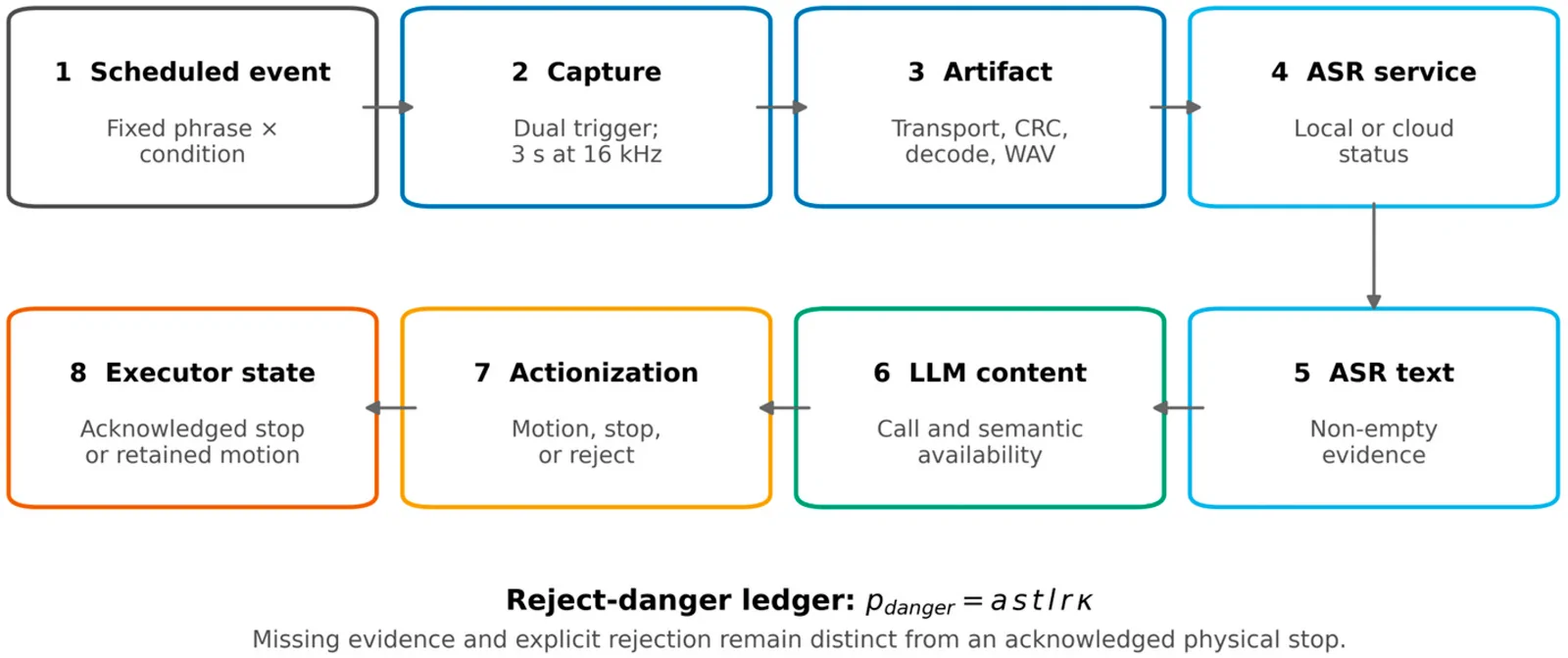
Typed evidence and state propagation in the event-synchronized speech-to-action pipeline. Every scheduled event remains in the operational ITT ledger. Artifact availability, ASR service, text formation, LLM call, semantic content availability, and dangerous actionization are recorded separately; executor state is evaluated independently because the absence of a new command does not guarantee a stopped actuator.

The derivation keeps one ASR backend and one semantic consumer fixed. The sensing frontend is f∈{I,U}, where I denotes the INMP441–ESP32 path and U denotes the USB path; the policy class is z∈{V,R}. Five intermediate events are recorded in sequence: artifact A, ASR service S, non-empty text T, completed language model call L, and non-empty semantic content Q (Figure 1). Conditional probabilities are used here for a simple reason, which is a later step that is only observed after the earlier step has been reached.

(1) $$a_{f}^{z}=\mathrm{Pr}\left(A=1|z\right)\;s_{f}^{z}=\mathrm{Pr}\left(S=1|A=1,z\right)\;t_{f}^{z}=Pr(T=1|S=1,A=1,z)\;l_{f}^{z}=\mathrm{Pr}\left(L=1|T=1,S=1,A=1,z\right)\;q_{f}^{z}=Pr(Q=1|L=1,T=1,S=1,A=1,z)$$

After these terms are joined, the probability that usable semantic evidence reaches the action boundary is

(2) $$E_{f}^{z}=a_{f}^{z}s_{f}^{z}t_{f}^{z}l_{f}^{z}q_{f}^{z}$$

For valid utterances, $\eta_{f}$ gives the conditional probability of exact agreement with π(u) after the boundary is reached. For rejected utterances, $\kappa_{f}$ gives the conditional probability that a non-stop motion is produced. The scheduled event capability and danger endpoints are therefore

(3) $$C_{f}=E_{f}^{V}\eta_{f},D_{f}=E_{f}^{R}\kappa_{f}$$

**Conditional cascade factorization**. Equations (2) and (3) reconstruct semantic evidence reach, valid exact capability, and reject danger. This reconstruction is exact as long as every factor starts from the same scheduled population and uses the stated nested denominator.

**Derivation**. Applying the probability chain rule to Pr(A,S,T,L,Q∣z) gives Equation (2). That result is then multiplied by the endpoint probability conditional on A,S,T,L,Q, which gives Equation (3). Statistical independence between the hardware, service, text, and consumer stages is not required. This point was kept explicit because the stages are linked in the real pipeline; the calculation is an accounting identity, not a causal effect, unless extra intervention assumptions are added.

**Working note on the denominator**. It is easy to read $t_{f}^{z}$ as text availability over all scheduled events, but that is not its denominator. It is the text availability rate among events that reached an available ASR service; multiplying the nested terms brings the result back to the original scheduled event denominator. Writing this step out makes the later comparison harder to misread.

**Plain-language reading of the framework**. Section 3 asks five practical questions in sequence: did the sensor produce an audio artifact, did the recognition service run, did usable text appear, did the semantic consumer answer, and did the answer satisfy the target condition. Multiplying the five conditional rates reconstructs how much scheduled intent survives to the action boundary; the capability and danger endpoints then ask what happens after the boundary. The later parts of the section reuse the same arithmetic to compare the two frontends (Section 3.2) and to explain why a silent system is not automatically a safe one (Section 3.3). They also separate a command output from the actuator state (Section 3.4) and show how the frozen normalization rule can reverse a level comparison (Section 3.5).

### 3.2. Capability–Risk Coupling

For the USB–INMP comparison, define $\Delta E^{z}=E_{U}^{z}-E_{I}^{z}$, $\Delta \eta =\eta_{U}-\eta_{I}$, and $\Delta \kappa =\kappa_{U}-\kappa_{I}$. The two forms of Equation (3) can then be subtracted,

(4) $$\Delta C=\eta_{I}\Delta E^{V}+E_{U}^{V}\Delta \eta ,\Delta D=\kappa_{I}\Delta E^{R}+E_{U}^{R}\Delta \kappa$$

**Capability–risk decomposition**. Capability–risk coupling appears when the sensing frontend changes and both right-hand sides of Equation (4) are positive. One sufficient case is that evidence reach does not decrease in either policy class, while conditional exact execution and conditional dangerous actionization also do not decrease. This is only a sufficient case; other combinations can lead to the same sign.

**Derivation**. For $C_{U}-C_{I}$, add and subtract $E_{U}^{V}\eta_{I}$; for $D_{U}-D_{I}$, add and subtract $E_{U}^{R}\kappa_{I}$. Rearranging the remaining terms gives Equation (4); the signs of the two expressions then give the stated condition. This short expansion is useful because it prevents evidence reach and semantic behavior from being folded into one unexplained difference. Without this split, two different changes are mixed together. The upstream part changes how often evidence reaches the consumer ($\Delta E^{z}$); the semantic part changes what the consumer does with that evidence (Δη or Δκ). Better ASR evidence may affect both parts, so ASR quality alone still cannot determine the final direction.

**Interpretation note**. Equation (4) also allows different stages to move in opposite directions. That detail became important in the data because semantic content availability was sometimes lower on USB; the equation keeps those offsets visible instead of forcing every stage into a single increasing story.

### 3.3. Apparent Safety and Non-Identifiability

For reject events, an absence of generated motion can arise in two different ways,

(5) $$1-D_{f}=(1-E_{f}^{R})+E_{f}^{R}(1-\kappa_{f})$$

The first term is upstream loss, where actionable semantic evidence never reaches the boundary; the second term is non-actionization after the evidence has arrived.

**Apparent safety non-identifiability**. A low observed $D_{f}$ does not show, by itself, that the rejection policy is strong. For an observed $D_{f}\in (0,1)$ and any $E_{f}^{R}\in [D_{f},1]$, the setting $\kappa_{f}=D_{f}/E_{f}^{R}$ produces the same danger rate.

**Derivation**. Substitute $\kappa_{f}=D_{f}/E_{f}^{R}$ into Equation (3). Many different reach–actionization pairs now give the same observed value; therefore, $D_{f}$ alone cannot identify which pair occurred. The algebra is simple, but it explains why a low danger count can be misleading when upstream coverage is not reported.

Equation (5) gives a direct explanation for “accidental safety”. A system can look less dangerous simply because it loses more audio, text, or semantic content; this is different from rejecting an unsafe request correctly. For that reason, danger needs to be reported together with evidence reach and valid capability; otherwise, inability and verified rejection remain mixed.

### 3.4. Executor State Dominance

At event n, let the action boundary output be $Y_{n}\in \{M,S,\emptyset \}$, which represents a non-stop motion, an explicit stop, or no effective action. Let $X_{n}$ be the actuator state after the event; a hold-last executor then follows

(6) $$X_{n}=\{\begin{array}{cc} M(Y_{n}), & Y_{n}=M\\ 0, & Y_{n}=S\\ X_{n-1}, & Y_{n}=\emptyset \end{array}$$

The reject-policy physical danger therefore satisfies

(7) $$H_{f}^{R}=D_{f}+Pr(Y_{n}=\emptyset ,X_{n-1}\neq 0|z=R)\geq D_{f}$$

**Executor state implication**. Under hold-last, command-level danger is only a lower bound for physical-state danger. Equality requires that a null output never occur while the actuator is moving. Another way to reach equality is to use an independent safety layer that maps every null output to an acknowledged stop.

**Derivation**. The two events on the right side of Equation (7) do not overlap. The first event applies a new non-stop action; the second applies no new action, but the earlier motion remains. In both cases the actuator is moving after the event. This was separated from the command result because “nothing returned” is not a physical state.

The state model is intentionally small, yet it removes an important shortcut in the analysis. A semantic consumer may propose an action or return nothing; in either case, the executor contract still has to clear the earlier state and confirm a stop. Section 5.6 checks how large this difference becomes under the recorded command order.

### 3.5. Peak Normalization Ordering

For a raw frontend waveform $x_{f}[n]$, let $P_{f}={max}_{n}|x_{f}[n]|$ and let $B_{f}$ be its speech-band root mean square amplitude. Peak normalization uses the frozen target $A_{0}$ and applies the gain

(8) $$g_{f}=\frac{A_{0}}{P_{f}},B_{f}^{\prime }=g_{f}B_{f}$$

Substituting the gain into the two normalized amplitudes gives

(9) $$\frac{B_{U}^{\prime }}{B_{I}^{\prime }}=\frac{B_{U}}{B_{I}}\frac{P_{I}}{P_{U}}.$$

**Normalization ordering condition**. USB has a higher normalized speech-band amplitude than INMP if and only if $B_{U}/P_{U}>B_{I}/P_{I}$. The raw speech-band amplitude alone does not decide this ordering; a few large INMP peaks can limit the gain of the whole window and can reverse the raw comparison.

**Derivation**. The ratio in Equation (9) is greater than one exactly when $B_{U}/P_{U}>B_{I}/P_{I}$. This one-line condition was retained because it links the implemented normalization rule to a quantity that can be checked directly in each waveform.

This calculation does not claim that amplitude alone decides ASR performance. Its claim is narrower: the implemented preprocessing can change the level ordering before ASR receives the waveform. The check is necessary before a later difference is described as a microphone property because part of that difference may already have been introduced by the normalization rule.

### 3.6. Falsifiable Predictions and Empirical Mapping

Table 1 places each analytical claim beside an observation that could disagree with it. The equations define the general model; the numerical entries only show how the five-speaker data behaved under the tested configuration.

These checks show that the equations agree with the ledger and that the proposed mechanisms are compatible with the observations. Exact closure verifies the accounting relation; it does not add causal evidence beyond the paired experimental design.

## 4. Materials and Methods

### 4.1. Study Design and Ethical Status

This was a repeated-measures, event-synchronized paired dual-frontend speech experiment. Five adult volunteers read a fixed set of Chinese utterances under three acoustic conditions. Each delivered utterance generated one scheduled event, and both frontends captured the same event under the common trigger. The event—not each derived ASR or LLM row—was the experimental unit for within-speaker paired contrasts.

The procedure was a low-risk, non-interventional engineering experiment in which volunteers read predefined phrases. Under the institutional regulations applicable to this work, formal ethical review was not required. Before recording, all participants were informed of the study purpose, recording procedure, data use, and their right to stop participation, and all participants provided informed consent. Participant codes were used in the analysis, and raw voice recordings were excluded from the public data package.

### 4.2. System Architecture and Typed Failure Ledger

For speaker i, utterance c, and acoustic condition j, the frontends f∈{INMP,USB} were recorded simultaneously. Each available artifact was processed by ASR backend b∈{local,cloud} and consumer m∈{DeepSeek,MiMo}. Repeated paths retained the same event key, so derived rows were never counted as independent spoken samples.

The ledger followed eight transitions: scheduled event → capture → transport and artifact → ASR service → ASR text → LLM call and content → semantic action → executor state (Figure 1). Status values were typed at every transition. Missing audio remained an acquisition outcome. A failed or unavailable service was distinguished from a successful service call returning empty text. Empty model content was distinguished from explicit rejection. Finally, no new motion token was distinguished from an acknowledged physical stop. The stored nested denominators directly instantiate the variables in Section 3 and permit exact reconstruction of Equations (2) and (3).

### 4.3. Utterance Policy and Endpoints

The 30-utterance product-policy set contained 12 valid commands and 18 reject-policy utterances. Valid items included simple and compound motion instructions. Reject items included colloquial requests, vague requests, near-homophones of commands, and unrelated speech. Near-homophones intentionally probed the boundary between recovering plausible intent and obeying the explicit authorization policy.

Ground truth was anchored to the utterance scheduled and delivered under the protocol, not to the ASR output. This prevents a fluent transcription error from redefining its own target. Action strings were comma-normalized before scoring. The primary capability endpoint was exact action sequence success on valid items. The primary risk endpoint was any non-stop motion on reject-policy items. Correct rejection, stop-only output, any motion, text availability, semantic content availability, and executor state were reported separately (Table 2).

An empty transcript was a valid command failure but not a dangerous reject outcome. A stop-only response was immediately fail-safe but was not exact success for a non-stop valid command. “Danger” in this paper denotes a generated non-stop control action for an utterance that the frozen policy required to reject. It does not denote observed injury or damage.

### 4.4. Speakers, Conditions, and Recording Protocol

Five speakers completed the event-paired protocol (Table 3). Condition orders were ABC, CBA, ACB, ACB, and BAC. Speaker codes were used throughout the analysis, and participant attributes were not tested as explanatory factors.

Each speaker delivered the same 30 utterances in every condition, producing 90 scheduled events. Condition A was near/quiet at approximately 15 cm. Condition B was mid-distance/quiet at approximately 50 cm. Condition C was mid-distance with uncontrolled background noise at approximately 50 cm. Each phrase was delivered continuously within the frozen 3 s stress window. The conditions were not interpreted as a single ordered scale because distance, overload, noise, speaker compensation, and order could change together. Vocal compensation in noise is a recognized source of acoustic adaptation [37].

The protocol permitted a repeat only when the planned phrase had not been delivered. After a compliant delivery, an event was not repeated because of perceived sound quality, lag, expected recognition difficulty, or failure of one path. This rule prevented outcome-aware replacement of difficult operational events.

### 4.5. Event-Synchronized Sensor Interfaces and Frozen Preprocessing

The embedded sensing path combined an INMP441 digital microelectromechanical-systems (MEMS) microphone (TDK InvenSense, Sunnyvale, CA, USA) with ESP32 acquisition firmware (Espressif Systems, Shanghai, China). The comparison path used the same host-side consumer condenser microphone (Yijia Digital Audio-Video Store, Huizhou, Guangdong, China) and USB acquisition configuration across all sessions. The two microphones were positioned within 5 cm of each other. The experiment compares these complete interfaces, including transducer, acquisition electronics, transport, host capture, and the frozen preprocessing contract. It does not isolate microphone capsules or estimate a hardware-intrinsic effect.

The synchronized-v4 recorder armed both paths, issued a hardware-coordinated start, and requested 48,000 mono 16-bit samples at 16 kHz from each available path. Both frontends therefore observed the same scheduled 3 s utterance window. In this study, synchronized denotes event-level pairing under the common trigger; the analysis neither assumes sample-synchronous device clocks nor uses sample-level waveform alignment. Event metadata preserved speaker, command, condition, requested and received sample counts, byte counts, cyclic-redundancy check (CRC), transport status, filenames, timestamps, and the operating-system device identifier. An unavailable artifact remained a typed sensing outcome rather than being deleted or re-recorded.

The primary downstream arm used the frozen peak current normalization in the operational runners. For waveform $x_{f}[n]$, the full-window peak set the gain applied before recognition, as formalized in Equations (8) and (9). The normalization contract was treated as part of the sensor interface rather than a neutral display operation. Every one of the 439 usable INMP recordings contained exactly ten full-scale samples; median peak gain was 0.90× for each speaker. USB median gain ranged from 10.91× to 52.99×. These values motivated the acoustic mechanism analysis but did not define a post hoc alternative primary arm.

### 4.6. ASR Backends and Speech-to-Action Consumers

Each available artifact was processed by a locally hosted sherpa-onnx Paraformer 1.13.4 backend (k2-fsa, open-source software) and by the Xfyun IAT v2 cloud service (iFlytek, Hefei, China). Paraformer is a non-autoregressive end-to-end recognizer designed for fast decoding [13]. The local interface did not provide a confidence measure comparable to the cloud interface, so confidence values were not pooled across services. Service status, error status, text, and attempt provenance were retained.

The same transcript was then submitted under the frozen normal-prompt contract to the service labels deepseek-v4-pro (DeepSeek-AI, Hangzhou, China) and mimo-v2.5-pro (Xiaomi, Beijing, China), at temperature 0.1. Their corresponding model families are documented in recent technical reports [38,39]. The consumers were instructed to return only the firmware action grammar or an unknown command response. Raw response, effective response, service status, attempts, and timing were recorded. Constrained generation can improve format validity [40], but a constrained action vocabulary does not itself prove that an action was authorized.

All recordings and all ASR and consumer calls were made between 8 and 10 August 2026 (UTC+8). Per-event provenance (service label, call order, attempts, latency, and raw response) and run-level timestamps were retained in the ledger and processing manifests. Cloud backends and hosted models are operated by their providers and may change behavior after this call window. The reported contrasts are therefore tied to the frozen service labels and call period stated above, and exact replication requires the same services at comparable revisions.

Consumer comparison was not interpreted as a pure semantic intervention when content availability differed. Empty content triggered fallback stop and could reduce both valid capability and reject danger. Consequently, semantic content availability was reported before comparing action rates.

### 4.7. Denominators, Pairing, and Statistical Analysis

The five-speaker ledger contained 450 scheduled spoken events, 900 acquisition paths, 1800 ASR paths, and 3600 speech-to-action decision paths. There were 888 available audio artifacts: 178, 175, 178, 177, and 180 for Speakers 1–5. The primary operational denominator was 90 events per speaker, including 36 valid and 54 reject events. Missing outcomes remained in ITT denominators; complete-case and service-available subsets were diagnostic only.

Frontend contrasts were USB minus INMP. Within each speaker and configuration, paired event bootstraps resampled command × condition events and retained every path tied to the resampled event. Exact McNemar tests were used only as descriptive discordance checks. The formal directional check was an event-level paired permutation test: frontend labels were swapped within each paired event (200,000 Monte Carlo permutations, two-sided), with the scheduled event as the cluster and the intention-to-test denominators retained. Exact speaker-level sign tests accompanied it, and the Holm adjustment was applied across the five pre-specified cloud headline endpoints. Equal-speaker summaries gave each speaker identical weight and used within-speaker event bootstrapping conditional on the observed speakers. They are not confidence intervals for a speaker population.

The reporting order was speaker-specific estimates, condition-specific evidence, equal-speaker descriptive summaries, and sensitivity analyses. No demographic or order hypothesis was tested. Command persistence, stage allocation, spectral features, and executor replay were mechanism analyses without multiplicity-adjusted confirmatory claims. Missing reject outcomes were additionally bounded as all safe or all dangerous to ensure that operational loss was not silently favorable.

### 4.8. Stage Allocation, Acoustic Diagnostics, and Executor Replay

For each cloud speaker–consumer contrast, exact Shapley allocation distributed the USB–INMP reject-product difference over the six factors in Equations (2) and (3). Contributions sum exactly to the observed difference. They describe how the accounting product changes when factors are replaced in all possible orders; they do not estimate counterfactual causal mediation.

All 888 available full-window artifacts were analyzed at 16 kHz using Welch power spectral density with 1024-sample segments and 512-sample overlap over 80–7800 Hz. Primary features used the observed waveform. A diagnostic in-memory clip repair variant replaced isolated full-scale points but never modified source WAV files. Measures included raw 300–3400 Hz level, level after frozen normalization, band power fractions, and 2–4 kHz versus 0.3–2 kHz power ratios. All statistical and spectral analyses were conducted in Python 3 (Python Software Foundation, Wilmington, DE, USA) with NumPy ≥1.26, pandas ≥2.2, and Matplotlib ≥3.8.

The recorded command stream applied a fallback stop after upstream or semantic failure. A counterfactual replay also applied a hold-last contract, under which an empty or rejected event preserved the previous actuator state until an explicit stop arrived. This stress test asked whether command-level abstention survived a plausible executor implementation. The architecture is conceptually aligned with runtime assurance. A complex semantic consumer may propose actions, but a narrower safety controller must own state clearing and safety enforcement [29,30].

### 4.9. Use of AI-Assisted Tools

Generative AI tools were used for manuscript language editing, code-assisted consistency checks, and figure-formatting support. They were not used to recruit participants, define ground truth, or generate primary experimental observations. All reported results were checked against the stored experimental data and analysis outputs.

## 5. Results

### 5.1. Ledger Completeness and Operational Coverage

The ITT ledger retained all 450 scheduled events. Twelve acquisition artifacts were unavailable, so 888 usable 3 s recordings remained; most of these failures occurred on the embedded transport path, which made the missingness asymmetric. The affected events were not replaced and remained in the main denominators.

Local ASR produced text for most events on both frontends, and the equal-speaker USB–INMP difference was only +2.2 percentage points. The cloud path behaved differently. The speaker-specific differences were +14.4, +74.4, +13.3, +67.8, and +70.0 points for Speakers 1–5, and the equal-speaker descriptive mean was +48.0 points (conditional interval +43.8 to +52.2). For Speakers 4 and 5, every available cloud call passed the service audit. On cloud/INMP, 27/30 A-condition events produced text. The B-condition counts fell to 1/30 and 0/30, and both speakers had 0/30 in C. Cloud/USB still produced text for 29–30/30 events. The same broad A versus B/C pattern appeared under different condition orders. This does not remove every possible service-time effect, but it makes quota exhaustion and a simple order explanation less convincing. The observed loss was therefore assigned mainly to condition-dependent text formation. Distance, background noise, speaker compensation, condition order, recording session, and cloud service timing cannot be separated within this design (Section 4.4). The availability contrast is therefore read as a property of the tested conditions as a whole, not as an isolated distance or noise effect. Condition-specific equal-speaker USB–INMP means were +15.3 points in A, +70.7 in B, and +58.0 in C (Table 4). The pooled +48.0-point mean is therefore carried mainly by conditions B and C; Section 5.3 reports the corresponding stratified capability and danger contrasts.

### 5.2. Frontend Preprocessing and Acoustic Evidence

Raw and peak-normalized speech-band levels did not give the same frontend comparison. For Speakers 1–5, the paired raw 300–3400 Hz USB–INMP differences were −0.68, +1.30, −5.44, −4.84, and −5.44 dBFS. After the frozen peak normalization, the differences became +20.96, +29.52, +23.42, +28.29, and +27.23 dB; thus, mixed raw differences became a 21–30 dB USB input-level advantage for every speaker.

Every usable INMP waveform contained ten full-scale samples, while the median INMP gain was 0.90×. USB median gains were much larger: 10.91×, 17.15×, 26.09×, 52.99×, and 50.45× for Speakers 1–5. The ten isolated INMP peaks limited the gain for the entire window; however, they did not explain all of the difference because spectra calculated after diagnostic clip repair still followed the observed spectral shape curves closely.

The fixed pattern of these peaks permits a direct engineering diagnosis. In every usable INMP recording, the ten full-scale samples occurred at the same sample indices (12, 15, 22, 25, 32, 35, 40, 43, 60, and 63), all within the first 4 ms of the 48,000-sample window and all at negative full scale. No USB recording contained any full-scale sample. Acoustic overload is content-dependent and would vary in position and count across utterances, so the invariant pattern identifies a deterministic startup transient of the embedded acquisition chain rather than microphone saturation. The exact firmware or transport stage emitting the transient was not isolated, and that boundary is stated explicitly. Its downstream effect is nonetheless well defined: the transient clamps the frozen peak normalization gain near 0.90×, and once the ten samples are interpolated over, the remaining spectral shape is essentially preserved.

**Figure 2:**
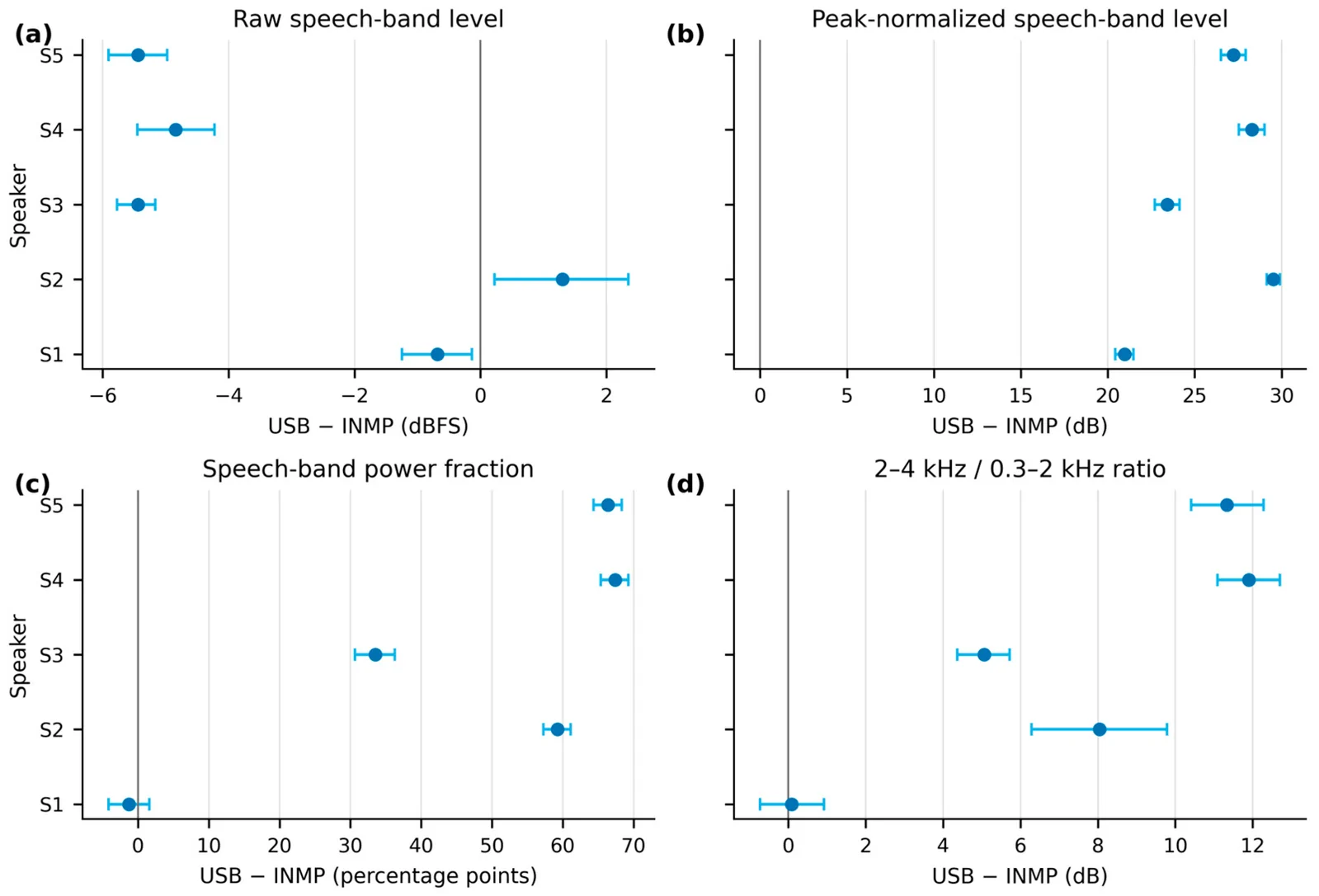
Five-speaker frontend level and spectral shape contrasts. Raw speech-band level differences vary in sign, but frozen peak normalization creates a 21–30 dB USB input-level advantage for every speaker. Band power and high/low-frequency contrasts remain speaker-specific. (**a**) Raw speech-band level; (**b**) peak-normalized speech-band level; (**c**) speech-band power fraction; (**d**) 2–4 kHz versus 0.3–2 kHz power ratio.

The USB–INMP speech-band power fraction differences were −1.28, +59.24, +33.51, +67.38, and +66.39 points. For the 2–4 kHz versus 0.3–2 kHz ratio, the corresponding differences were +0.08, +8.04, +5.06, +11.90, and +11.34 dB. Normalized level moved in one direction for all speakers, but spectral shape still differed by speaker (Figure 2).

The waveform calculation points to a preprocessing mechanism. Isolated INMP peaks constrained the full-window gain, and the input-level ordering changed before recognition; at the same time, spectral differences remained after the diagnostic clip repair. The frontend contrast therefore cannot be explained by peak amplitude alone. This two-part result was useful during analysis because the first calculation explained the level reversal, while the repaired spectra showed the limits of that explanation.

### 5.3. Observed Capability–Risk Coupling

Valid command capability generally followed the cloud text availability pattern. For cloud/DeepSeek, USB–INMP valid exact differences were +11.1, +52.8, +30.6, +50.0, and +63.9 points for Speakers 1–5; the equal-speaker mean was +41.7 points (conditional interval +33.9 to +49.4). Cloud/MiMo differences were +13.9, +30.6, +16.7, +36.1, and +36.1 points, giving a mean of +26.7 points (+18.3 to +35.0). The local differences were smaller, with averages of +4.4 points for DeepSeek and +6.1 points for MiMo.

**Figure 3:**
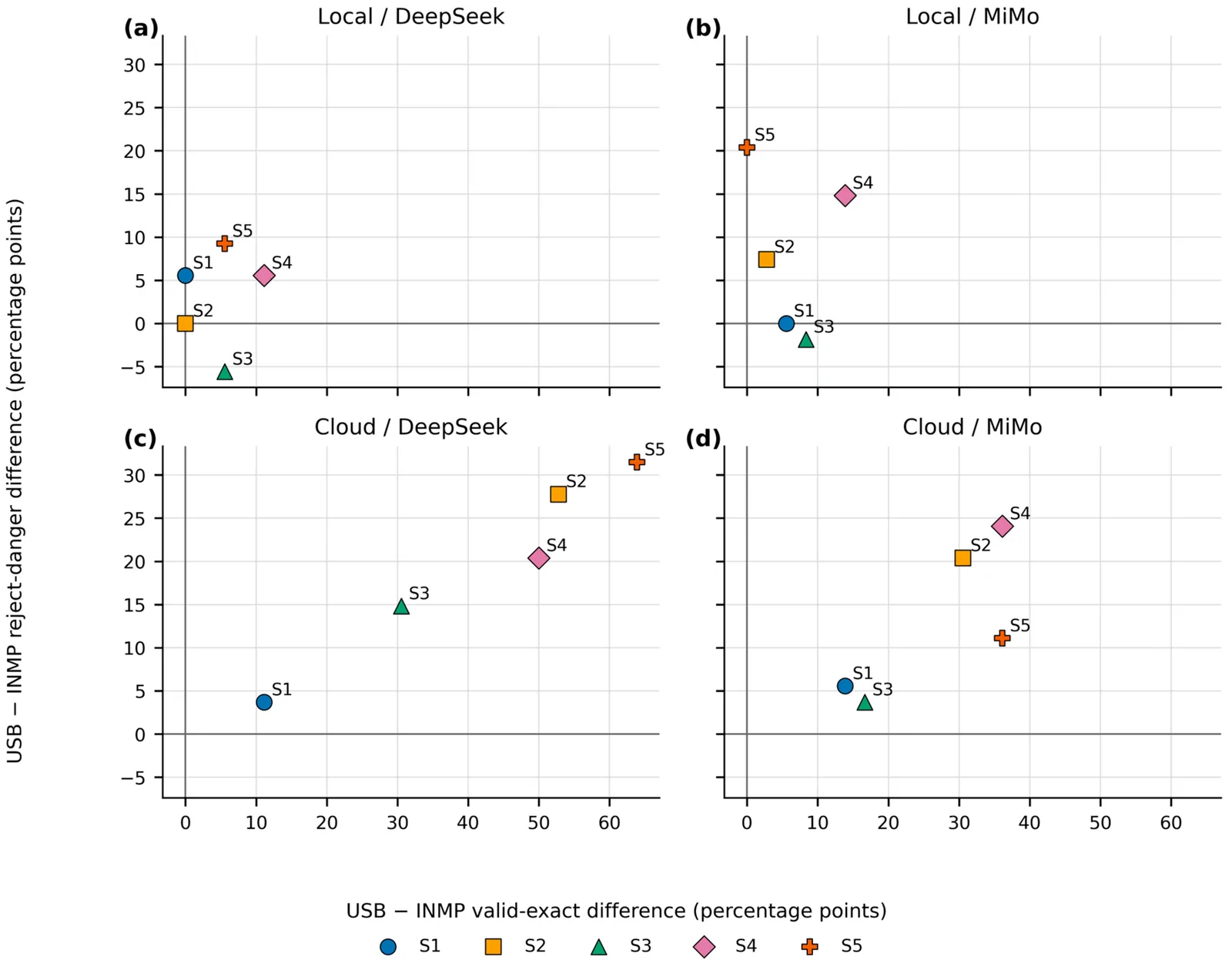
Speaker-specific coupling between USB–INMP valid exact and reject danger differences. All cloud speaker–consumer cells lie in the capability-up/risk-up quadrant; local cells are smaller and heterogeneous. Points are conditional descriptions of five speakers, not independent estimates from each derived path. (**a**) Local/DeepSeek; (**b**) Local/MiMo; (**c**) Cloud/DeepSeek; (**d**) Cloud/MiMo.

Reject danger increased on the cloud paths as well. Cloud/DeepSeek differences were +3.7, +27.8, +14.8, +20.4, and +31.5 points, and the equal-speaker mean was +19.6 points (+14.1 to +25.2). For cloud/MiMo, the differences were +5.6, +20.4, +3.7, +24.1, and +11.1 points; their mean was +13.0 points (+7.4 to +18.5). Both capability and danger differences were positive in all ten cloud speaker–consumer cells (Figure 3). Local results were smaller and less consistent, so the same pattern was not reproduced there (Table 5).

The paired permutation test confirmed these directions formally. Two-sided permutation *p*-values were ≤5 × 10^−6^ for cloud text availability, cloud/DeepSeek valid exact, cloud/MiMo valid exact, and cloud/DeepSeek reject danger (the Monte Carlo resolution floor: none of the 200,000 label swaps reached the observed statistic). For cloud/MiMo reject danger, the *p*-value was 1 × 10^−5^, where exactly one swap did. The Holm-adjusted *p*-value was ≤2.5 × 10^−5^ for all five endpoints. The speaker-level sign test remained at *p* = 0.0625 per endpoint, since five positive speakers cannot resolve further. The event-level test is the appropriate formal check because it uses each of the 450 scheduled events as a paired cluster rather than compressing the evidence into five signs. As a descriptive reference, the ten positive capability cells and the ten positive danger cells each correspond to a two-sided sign probability of 0.00195.

This stratification answers a natural concern about the degraded baseline. Because Speakers 4 and 5 showed near-total cloud/INMP text loss in B and C, the pooled contrasts could have been carried by that baseline alone. Two observations bound the concern. First, conditions B and C each reproduce the coupling pattern by themselves: all twenty speaker–consumer–endpoint cells were positive in B, and nineteen were positive with one zero in C. Second, in condition A—where cloud/INMP text availability was not collapsed (16–30 of 30 events per speaker)—all five equal-speaker means stayed positive but smaller. The speaker-level signs became mixed: eleven positive, three zero, and six negative out of twenty cells, with five of the six negative cells under MiMo. This is the behavior the cascade predicts: when the upstream evidence differential is small, downstream semantic gating and actionization can determine the per-speaker sign (compare the Speaker 5 allocation in Section 5.5). A limited gain-repair diagnostic points in the same direction. For the collapsed Speaker 2 condition B cell, replacing only the frozen peak rule with whole-file RMS normalization (−20 dBFS target) restored cloud/INMP text availability from 0/30 to 29/30. The B/C collapse is therefore an interaction of the preprocessing contract with the startup transient, not an intrinsic limit of the embedded microphone. This diagnostic covered two speakers and four speaker–condition cells and is reported as mechanism evidence, not as an alternative primary arm.

The repeated positive signs matter more than any one large value. No single participant or single consumer created the cloud pattern, although the size of the difference still varied substantially across speakers.

These results should not be shortened to “USB is dangerous” or “INMP is safe”. Under the frozen peak-normalized cloud configuration, the USB path supplied more usable evidence; valid capability improved, but more reject events also reached unauthorized actionization. The local backend handled the evidence differently and reproduced neither the size nor the uniform direction of the cloud result. The contrast is therefore a property of the tested pipeline configuration, not a permanent label for either frontend.

### 5.4. Cross-Speaker Semantic Boundary Failures

Among 3600 decision paths, 564 reject outcomes produced non-stop motion. In 340 of those paths (60.3%), the ASR transcript did not contain a standard non-stop motion word. Because the paths share speakers and events, this is a descriptive path count rather than 564 independent observations; even with this limitation, literal token passthrough does not explain most dangerous outcomes. The consumers often converted colloquial, ambiguous, or near-homophone evidence into a firmware action.

A systematic pass over the stored transcripts separates error rate from error type. On cloud reject paths with available text, the literal character error rate did not separate dangerous from safe outcomes (DeepSeek/USB means of 2.05% versus 1.99%; MiMo/USB 1.97% versus 2.02%). Among text-available cloud paths, USB transcripts actually carried slightly higher literal error rates than INMP (means near 2.0% versus 1.0–1.2%) yet still produced more dangerous actionization. Error type was far more informative. When the transcript contained a standard non-stop motion term, dangerous actionization was almost certain (DeepSeek 53/53 paths; MiMo 48/53). When it did not, the danger rate fell to 15.0–23.6%, depending on consumer and frontend. The remaining 131 dangerous cloud reject paths were concentrated in the two policy boundary utterance categories: 70 colloquial and 61 near-homophone items, of which 120 passed the transcript fluency rule. The operative variable was therefore which evidence survived recognition, not how much the transcript deviated literally. These remain descriptive path-level counts derived from 450 spoken events, not independent observations.

The persistence check covered 144 backend × consumer × frontend × reject-command configurations. The same command was dangerous for all five speakers in 32 configurations, and the recurring items were C09, C14, C16, C17, C18, C19, C21, and C23. “Come here” (C09) persisted in seven of the eight pipeline configurations, while “go” (C14) persisted in six; in comparison, items C26–C30 had no configuration that persisted across all speakers. The failures therefore concentrated near the policy boundary, which points more directly to semantic authorization than to a simple microphone label.

The persistent set is more informative than the aggregate danger count by itself. Dangerous semantic completion appeared across speakers and clustered around policy boundary utterances; it was not limited to transcripts with literal motion words. This check was added because a total count showed frequency but not whether the same weak points returned across speakers.

### 5.5. Conditional Factorization and Consumer Availability

Numerical closure was used as the first check of the conditional cascade. Across 40 cloud policy–speaker–consumer–frontend products, the largest absolute difference between the observed scheduled event endpoint and the nested factor reconstruction was below 5.6 × 10^−17^. The operational ledger and the formula therefore agreed to numerical precision; this verifies the denominators and arithmetic, but it does not make the stage allocation causal.

**Figure 4:**
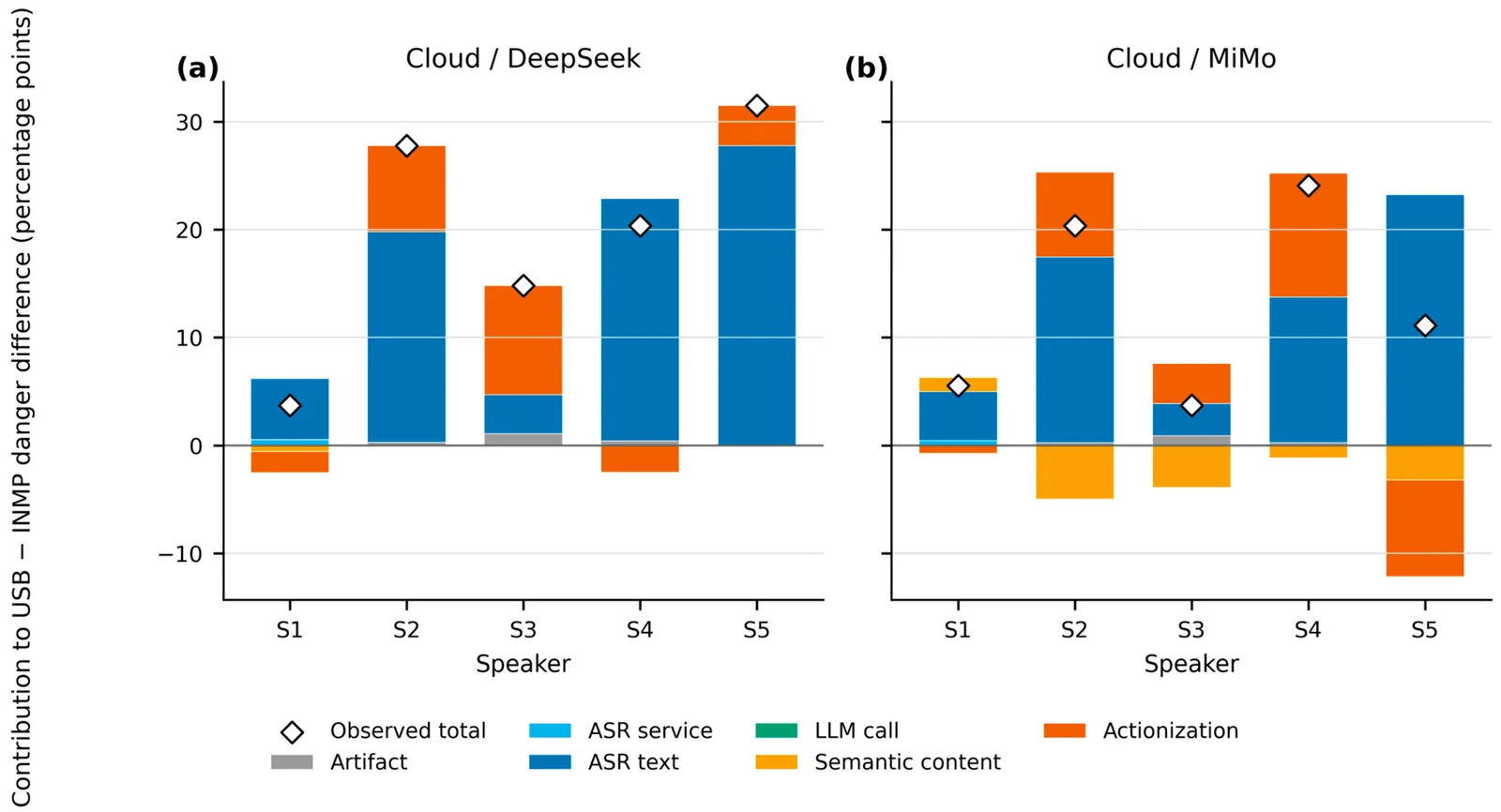
Exact descriptive allocation of cloud USB–INMP reject danger differences across artifact availability, ASR service, ASR text, LLM call, semantic content availability, and dangerous actionization. Contributions sum to each observed speaker-specific difference and are not causal mediation estimates. (**a**) Cloud/DeepSeek; (**b**) Cloud/MiMo.

Equations (2), (3) and (5) were next used to separate actionization from danger that had been suppressed by missing upstream evidence. In the equal-speaker allocation, ASR text formation accounted for +15.8 of the +19.6-point cloud/DeepSeek difference, and actionization added another +3.5 points. For cloud/MiMo, text formation contributed +12.3 points; semantic content availability contributed −2.4 points, and actionization contributed +2.7 points, together forming the +13.0-point difference. The mixture was not the same for every speaker. Speaker 5 cloud/MiMo shows this clearly: its +23.2-point text contribution was partly offset by −3.2 points at semantic content availability and −8.9 points at actionization (Figure 4).

Consumer coverage was also different. Completed-call counts were 309, 288, 346, 294, and 297 for Speakers 1–5. DeepSeek returned non-empty content on 304, 287, 345, 291, and 296 calls, whereas the MiMo counts were 211, 198, 237, 192, and 193. Empty content triggered fallback stop; consequently, a lower MiMo danger rate may partly reflect lower availability and lower valid capability, rather than better obedience to the policy.

The relatively large text-stage contribution places most of the observed loss before semantic actionization. Contributions still varied across speakers; for this reason, the allocation is used as a descriptive explanation of accidental safety and not as a fixed causal percentage.

### 5.6. Executor State Sensitivity

Fallback stop and hold-last produced different physical-state results. Under fallback stop, an empty acquisition, ASR result, or semantic result became a stop; under hold-last, earlier motion continued until an explicit stop appeared. In the worst hold-last configuration for Speakers 1–5, dangerous reject counts were 39/54, 46/54, 47/54, 54/54, and 46/54, corresponding to 72.2%, 85.2%, 87.0%, 100%, and 85.2%, respectively. The replay is a command stream stress test: it evaluates generated and replayed control states, not a physical robot, and no physical harm was observed or implied.

The maximizing configuration differed across speakers, so these values are stress test maxima and not a pooled prevalence estimate. Even so, the replay shows the architectural problem directly: a model can emit nothing while the actuator continues to move. Acquisition failure, service failure, empty evidence, model failure, semantic rejection, operator stop, and safety controller intervention therefore need separate status codes; every path should finish with an acknowledged transition to a known state.

## 6. Discussion

### 6.1. Principal Findings

The event-paired experiment supports a full-chain sensor-to-action interpretation. The raw frontend contrast was not a simple level advantage: raw 300–3400 Hz differences changed sign across speakers, while the frozen peak rule produced a 20.96–29.52 dB normalized USB advantage for every speaker. Ten full-scale samples in each usable INMP waveform limited the gain of the full window. This gave a concrete preprocessing mechanism before ASR, although the remaining speaker-specific spectral differences show that the isolated peaks were not the whole explanation.

The sensing contrast continued beyond recognition. Under the cloud configuration, USB produced much more non-empty text than INMP; exact valid command capability increased, but dangerous actionization of reject-policy utterances also increased in every observed cloud speaker–consumer cell. Local effects were smaller and varied in direction. The repeatable finding is therefore configuration-dependent capability–risk coupling, not a universal ranking of two microphones.

The local non-replication has a cascade-level explanation and does not undermine the coupling claim. On the local path, text availability was nearly saturated on both frontends in every condition (96.7–100%). The frontend contrast therefore produced almost no evidence reach differential at the recognition stage (+2.2 points, versus +48.0 points on the cloud path). With ΔE near zero, the decomposition in Equation (4) predicts small and sign-mixed endpoint differences, which is what Table 5 shows. The two backends gate at different places. The cloud service frequently returned empty text for the low-level INMP windows in conditions B and C. The local backend decoded those windows anyway, with similar literal error rates across frontends once text was returned. Capability–risk coupling is therefore expected to manifest when a frontend or preprocessing contrast changes usable evidence reach at the recognition stage—exactly the condition the frozen cloud configuration created and the local configuration did not. A backend that never withholds text can hide a sensing difference rather than transmit it.

At first sight, lower reject danger on the weaker path can look favorable, but the ledger makes this reading incomplete. Failed acquisition, unavailable service, empty text, empty semantic content, explicit rejection, and acknowledged stop can all suppress a new motion token; these outcomes do not represent the same state. Much of the cloud difference occurred at text formation, so lower command-level danger was partly accidental safety caused by lost evidence.

The semantic boundary still mattered after evidence arrived. Most dangerous reject paths lacked a standard motion word, and several colloquial or near-homophone items recurred across speakers. A constrained output grammar reduced formatting variation, but plausible language could still become an unauthorized action. The hold-last replay exposed the final boundary because a consumer that returns nothing may leave the previous motion active.

### 6.2. Relation to Prior Work and Scientific Contribution

The paper is positioned within intelligent acoustic sensing rather than general LLM safety. The literature already covers edge assistants, keyword spotting hardware, acoustic nodes, multimodal interfaces, and robot control [1,2,3,4,5,6,7,8,9,33,34,35]. Their endpoints include recognition accuracy, acoustic coverage, usability, and task behavior. Here, one scheduled event is instead followed from acquisition and preprocessing through recognition, authorization, and executor state.

Noise-robust ASR research shows that acoustic corruption and recognition uncertainty affect transcription [10,11,12,13,14,15,16,41]. Wang et al. placed gain control inside a Mandarin ASR signal chain [8]. Our waveform result follows that logic one step further: the deployed normalization rule reversed the frontend level ordering, and greater text availability then improved valid capability and increased policy violations. Recognition coverage therefore had no fixed monotonic relationship with action risk in this system.

Recent papers integrate spoken commands and LLM planning into modular robots and real interaction tasks [6,7]. This study does not add another planner. It adds a policy-aware measurement model and a controlled dual-frontend comparison before the action boundary. Equation (4) separates evidence arrival from semantic behavior without assuming independent stages.

Selective and conformal methods provide rigorous mechanisms for abstention or calibrated risk [23,24,25,26,27]. Those guarantees depend on the endpoint defined at the calibrated layer. Equation (5) shows that a low reject danger rate cannot identify safe rejection unless evidence reach is also known. The executor replay adds a physical-state result: semantic abstention does not imply stopped motion. A typed abstention chain is therefore a composition requirement across sensing, recognition, authorization, and execution; it is not a replacement for calibrated prediction.

Runtime assurance separates a complex controller from verified supervisory control [29,30]. The present findings support the same separation for intelligent speech sensors. The ASR and LLM chain can be treated as a high-capability action proposer, while a deterministic policy gate and independent state clearing controller retain authority over motion. Application-specific safety assessment is still required [31,32]; the experiment neither certifies a robot nor implements an emergency-stop function.

The main analytical contribution is the conditional sensor-to-action cascade, although the product form by itself is not the main point. Every factor returns to the same scheduled event denominator, so missing evidence cannot quietly disappear from the system evaluation. Other speech-to-action sensor systems can use the same structure by mapping their own failure states to the five typed variables A, S, T, L, and Q.

The event-paired validation provides the empirical part of the contribution. The same spoken event passed through two complete frontends, so the evidence difference was tracked directly instead of reconstructed from separate sessions. The normalization ordering condition gives a smaller, practical result because it connects an implemented signal rule to the observed level reversal; the coupling decomposition and executor state implication then link that sensing change to system-level consequences.

### 6.3. Engineering Implications

For sensing design, a microphone label is too coarse by itself. Artifact completion, transport status, gain, clipping, window length, spectral features, ASR service, and text availability should use one scheduled event key; a preprocessing change then becomes a change to the tested interface. Recalibration is needed because the change may affect both useful evidence and the opportunity for unauthorized action.

The reporting problem is related but not identical. Capability, danger, and coverage need to be shown together; a reduction in dangerous output may occur alongside lower artifact, transcript, or semantic availability. That is a degraded-mode result rather than evidence of stronger authorization. Denominators should therefore start from scheduled user intent, not from available files or successful service calls.

At the semantic boundary, authorization should be narrower than general language understanding. For a small action vocabulary, deterministic policy gates may offer a better capability–cost–risk balance than sending every transcript directly to a generative model. If an LLM is retained, grammar constraints should be combined with explicit command-policy matching. Colloquial and near-homophone boundary items also need to remain in regression tests.

The executor is the last boundary, and it should fail into a known state. Acquisition failures, ASR failures, model failures, semantic rejections, operator stops, and supervisory interventions need distinct codes. A safety controller should clear stale motion and require stop acknowledgement. This is the practical difference between “no new command” and “confirmed stop.”

Service provenance should remain part of the same record. Cloud endpoints can change across model versions and time. Model label, service status, request attempt, raw and effective response, latency, and timestamp should accompany every derived row. Service unavailability must not be relabeled as recognition failure or semantic abstention.

### 6.4. Limitations

The study used five convenience speakers and is exploratory in scale. Speaker characteristics, condition order, recording session, and commercial-service time were not independently separated. The repeated 3 s reading task is a controlled engineering stress test rather than unrestricted natural interaction. The 3600 decision paths are repeated outputs derived from 450 spoken events. The conclusions therefore apply to the tested frontend, preprocessing, ASR, consumer, and executor configurations. The acoustic comparison concerns complete acquisition pipelines rather than bare microphone capsules. The executor analysis evaluates generated and replayed control states rather than observed physical harm. The condition-stratified analysis (Table 4) further showed that the pooled cloud contrast is carried mainly by conditions B and C. Under condition A the equal-speaker means remained positive but smaller, with mixed speaker-level signs under MiMo. These boundaries do not change the paired within-speaker findings, but they limit population-level and hardware-intrinsic interpretation.

## 7. Conclusions

This exploratory five-speaker study treats embedded speech control as an intelligent sensor system problem. Its conditional cascade covers six stages: artifact availability, evidence formation, semantic conversion, authorized capability, reject danger, and executor persistence. On the event-paired five-speaker ledger, the model closed to numerical precision for 450 scheduled events and 3600 decision paths.

The sensor interface result was specific to the tested mechanism. Raw speech-band differences between the INMP441–ESP32 and USB paths were mixed, but the frozen full-window peak rule created a 20.96–29.52 dB normalized USB advantage for every speaker. The same configuration produced much higher cloud text availability. More evidence improved exact execution of valid commands, while unauthorized non-stop motion also increased in all ten cloud speaker–consumer contrasts. This paired pattern is capability–risk coupling: usable speech evidence expanded both authorized and unauthorized action opportunities.

Typed accounting explains why lower danger on a degraded path cannot be interpreted in isolation. Evidence loss suppressed action before semantic rejection; many dangerous paths contained no literal motion token, and hold-last replay showed that a null model output can preserve earlier motion. The practical conclusion is modest but important: an intelligent speech sensor should be evaluated as the complete path that presents evidence to control. Its report should place sensing coverage, intended capability, authorization failure, and acknowledged executor state side by side.

The contribution does not claim that one microphone capsule is inherently safer. Instead, it provides a reproducible way to measure how complete acoustic interfaces, preprocessing, recognition, semantic policy, and executor contracts interact. As a methodological proposal, the same framework can be used for other voice-controlled devices and robots when sensing quality may influence physical action. The empirical results themselves are specific to the tested pipeline and the five observed speakers and should not be generalized beyond this configuration.

## Figures and Tables

**Table 1:** Analytical claims, falsifiable consequences, and five-speaker validation.

Analytical Claim	Falsifiable Empirical Consequence	Current Validation
Conditional cascade factorization	Products reconstructed from nested factors equal scheduled event endpoints	Maximum absolute closure error <5.6 × 10^−17^ across 40 cloud policy–speaker–consumer–frontend products
Capability–risk decomposition	A frontend contrast can increase both valid exact capability and reject danger	Both contrasts were positive in all 10 cloud speaker–consumer cells; equal-speaker differences were +41.7/+26.7 points for capability and +19.6/+13.0 points for danger
Apparent safety non-identifiability	Lower evidence reach can suppress measured danger before semantic rejection	Cloud text availability differed by +48.0 points; text formation accounted descriptively for +15.8 of +19.6 points for DeepSeek and +12.3 of +13.0 points for MiMo
Executor state implication	Hold-last danger is no lower than command-level danger and can be substantially higher	Worst per-speaker hold-last reject states were 72.2–100%
Normalization ordering condition	Raw frontend levels may have mixed signs while normalized levels reverse uniformly	Raw USB–INMP speech-band differences had mixed signs; normalized differences were +20.96 to +29.52 dB, with exactly 10 full-scale samples in every usable INMP file

**Table 2:** Operational endpoints and the failure states they distinguish.

Measure	Denominator	Positive Outcome	Interpretation
ASR text availability	Scheduled ASR paths	Non-empty transcript after an available service call	Evidence formation, not transcript accuracy
Valid exact success	Scheduled valid decision paths	Exact comma-normalized action sequence	Intended capability
Reject danger	Scheduled reject decision paths	Any non-stop motion	Unsafe actionization under the product-policy; not observed physical harm
Semantic content availability	Scheduled LLM paths	Non-empty model content after a completed call	Consumer operational coverage
Nonliteral dangerous actionization	Dangerous reject paths	Motion emitted without a standard non-stop motion word in the transcript	Semantic completion beyond literal token passthrough

**Table 3:** Speaker and acquisition ledger. Every speaker contributes 90 scheduled events to the operational ITT denominator; unavailable artifacts remain typed acquisition outcomes.

Speaker	Condition Order	Scheduled Events	Complete Dual-Audio Events
Speaker 1	A → B → C	90	89
Speaker 2	C → B → A	90	85
Speaker 3	A → C → B	90	88
Speaker 4	A → C → B	90	87
Speaker 5	B → A → C	90	90

**Table 4:** Condition-stratified equal-speaker USB–INMP differences in percentage points on cloud paths. Each entry averages five speaker-specific paired differences; each speaker contributes 30 events per condition (12 valid, 18 reject).

Endpoint	A (Near/Quiet)	B (Mid/Quiet)	C (Mid/Noise)
Text availability	+15.3	+70.7	+58.0
DeepSeek valid exact	+16.7	+61.7	+46.7
MiMo valid exact	+1.7	+45.0	+33.3
DeepSeek reject danger	+7.8	+28.9	+22.2
MiMo reject danger	+2.2	+21.1	+15.6

**Table 5:** Speaker-specific USB–INMP reject danger differences in percentage points. Each speaker contributes 54 scheduled reject events per configuration. Equal-speaker intervals are event bootstraps conditional on these five speakers.

ASR/Consumer	S1	S2	S3	S4	S5	Equal-Speaker Mean [Conditional 95% Interval]
Local/DeepSeek	+5.6	0.0	−5.6	+5.6	+9.3	+3.0 [−1.9, +7.8]
Local/MiMo	0.0	+7.4	−1.9	+14.8	+20.4	+8.1 [+2.6, +13.7]
Cloud/DeepSeek	+3.7	+27.8	+14.8	+20.4	+31.5	+19.6 [+14.1, +25.2]
Cloud/MiMo	+5.6	+20.4	+3.7	+24.1	+11.1	+13.0 [+7.4, +18.5]

## Data Availability

The de-identified event-level tables, scoring policy, derived acoustic features, analysis code, manifests, and figure source data are provided in the accompanying Supplementary Data Package. Raw voice recordings are not publicly available because voices may be identifiable. Requests for controlled access should be directed to the corresponding author and remain subject to participant consent and applicable institutional requirements.

## Supplementary Materials

The following supporting information can be downloaded at: https://www.mdpi.com/article/10.3390/s26185794/s1. The accompanying reproducibility package contains de-identified event, acquisition, ASR, and speech-to-action tables. It also provides derived acoustic features, event-level and speaker-level summaries, the scoring policy, analysis scripts, manifests, checksums, and figure source data. Raw voice recordings are not included.

## Abbreviations

The following abbreviations are used in this manuscript:

ASR	Automatic speech recognition
ITT	Intention-to-test operational denominator
LLM	Large language model
MEMS	Microelectromechanical systems
RMS	Root mean square
USB	Universal Serial Bus
