# Supplementary material for: Preprocessing and Capability–Risk Coupling in an Event-Synchronized Dual-Frontend Speech Sensor System
Source: Sensors (Basel). 2026 Sep 12;26(18):5794. doi: 10.3390/s26185794 (PMC13611701; doi:10.3390/s26185794)
Supplement: Supplementary file 1 [file sensors-26-05794-s001.zip › figures/figure_1_typed_failure_chain.pdf]

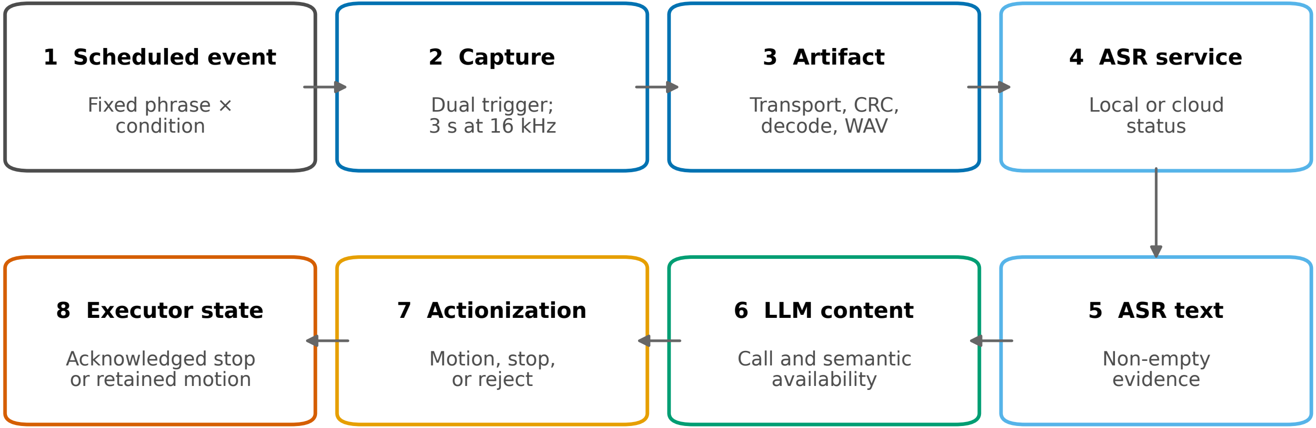

Reject-danger ledger: $p_{danger} = a\,s\,t\,l\,r\,\kappa$

Missing evidence and explicit rejection remain distinct from an acknowledged physical stop.
