# Supplementary material for: Preprocessing and Capability–Risk Coupling in an Event-Synchronized Dual-Frontend Speech Sensor System
Source: Sensors (Basel). 2026 Sep 12;26(18):5794. doi: 10.3390/s26185794 (PMC13611701; doi:10.3390/s26185794)
Supplement: Supplementary file 1 [file sensors-26-05794-s001.zip › figures/figure_2_frontend_spectral_contrasts.pdf]

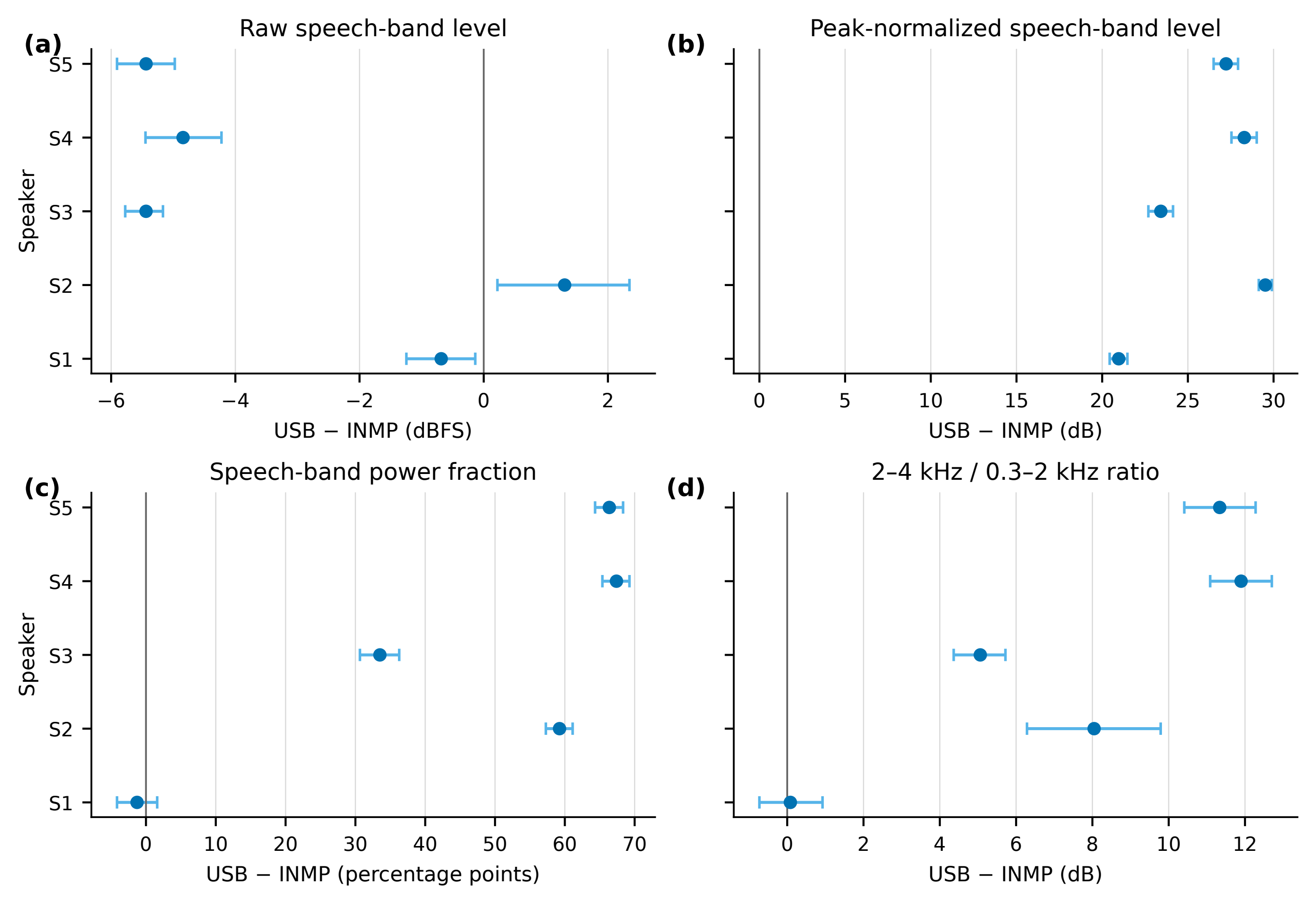
(a)
Raw speech-band level
S5
S4
S3
S2
S1
Speaker
−6
−4
−2
0
2
USB − INMP (dBFS)
(b)
Peak-normalized speech-band level
0
5
10
15
20
25
30
USB − INMP (dB)
(c)
Speech-band power fraction
0
10
20
30
40
50
60
70
USB − INMP (percentage points)
(d)
2–4 kHz / 0.3–2 kHz ratio
0
2
4
6
8
10
12
USB − INMP (dB)
