# Supplementary material for: Preprocessing and Capability–Risk Coupling in an Event-Synchronized Dual-Frontend Speech Sensor System
Source: Sensors (Basel). 2026 Sep 12;26(18):5794. doi: 10.3390/s26185794 (PMC13611701; doi:10.3390/s26185794)
Supplement: Supplementary file 1 [file sensors-26-05794-s001.zip › figures/figure_3_capability_risk_coupling.pdf]

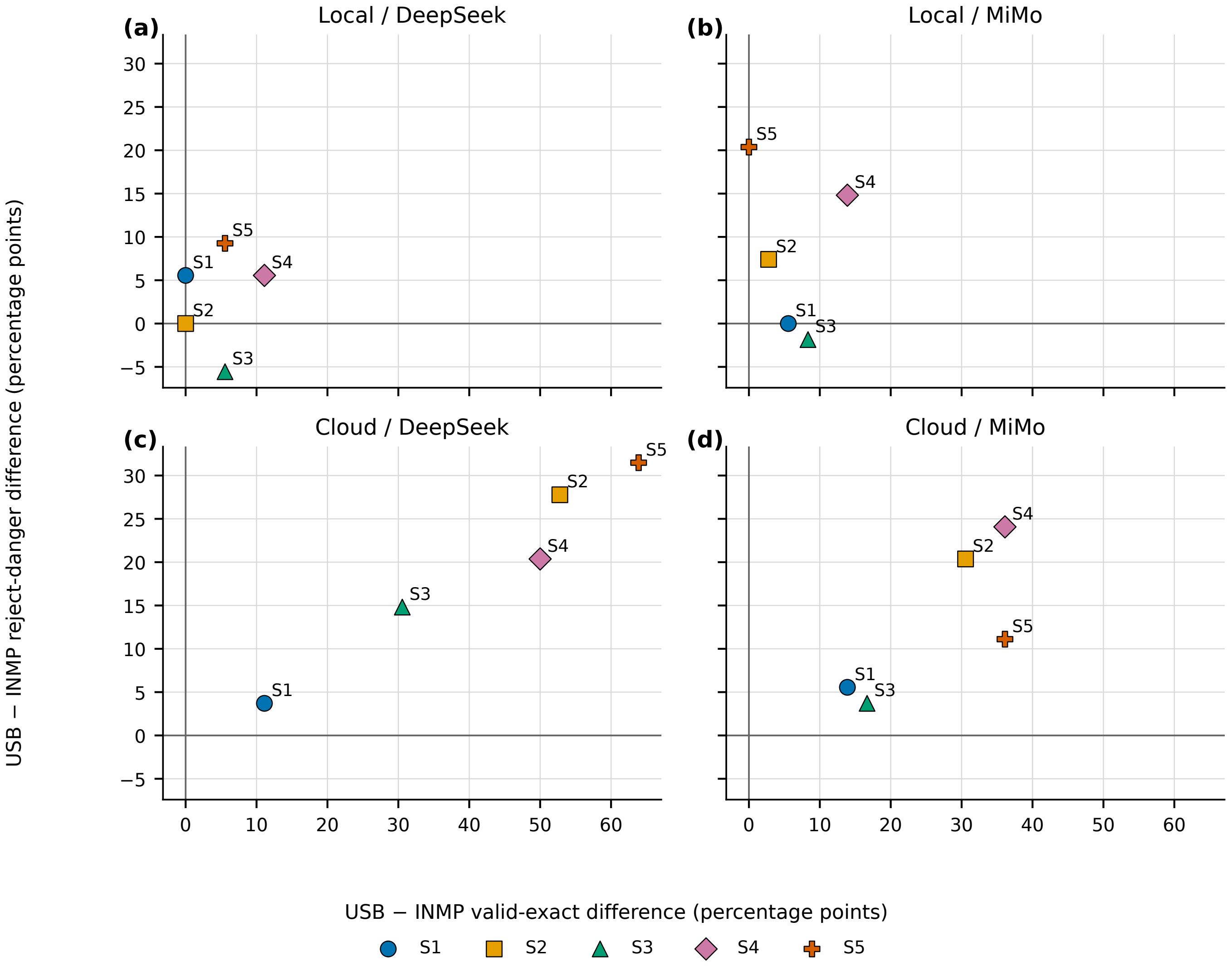

(a)
Local / DeepSeek
(b)
Local / MiMo
(c)
Cloud / DeepSeek
(d)
Cloud / MiMo
S1
S2
S3
S4
S5
30
25
20
15
10
5
0
−5
0
10
20
30
40
50
60
USB − INMP reject-danger difference (percentage points)
USB − INMP valid-exact difference (percentage points)
