# Supplementary material for: Preprocessing and Capability–Risk Coupling in an Event-Synchronized Dual-Frontend Speech Sensor System
Source: Sensors (Basel). 2026 Sep 12;26(18):5794. doi: 10.3390/s26185794 (PMC13611701; doi:10.3390/s26185794)
Supplement: Supplementary file 1 [file sensors-26-05794-s001.zip › figures/figure_4_cloud_stage_allocation.pdf]

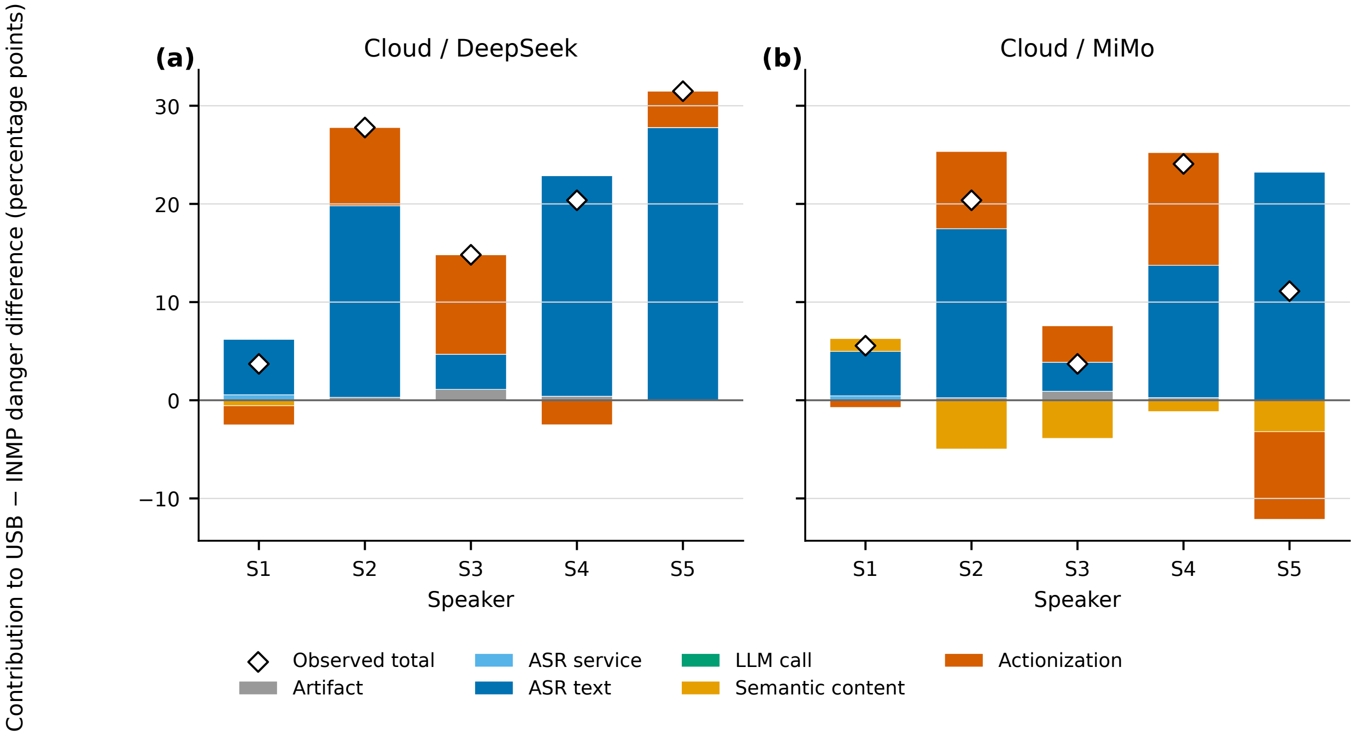

Contribution to USB − INMP danger difference (percentage points)
(a)
Cloud / DeepSeek
(b)
Cloud / MiMo
30
20
10
0
−10
S1
S2
S3
S4
S5
Speaker
Observed total
Artifact
ASR service
ASR text
LLM call
Semantic content
Actionization
